# Coping With the Crash: Intraoperative Management of Ventricular Wall Rupture During Coronary Artery Bypass Graft Surgery

**DOI:** 10.7759/cureus.75060

**Published:** 2024-12-03

**Authors:** Emily Bergbower, Reney Henderson

**Affiliations:** 1 Anesthesiology, University of Maryland Medical Center, Baltimore, USA

**Keywords:** cardiac surgery, cardiothoracic anesthesia, emergent cardiopulmonary bypass, left ventricular wall rupture, refractory shock

## Abstract

Left ventricular (LV) free wall rupture is a rare and often fatal complication of an acute myocardial infarction. We report the case of an LV free wall rupture after the induction of general anesthesia in an elderly woman who presented for a coronary artery bypass graft (CABG) procedure in the setting of an inferior wall ST elevation myocardial infarction (STEMI) four days prior. This case emphasizes both the differential diagnosis for and the management of refractory hypotension. Additionally, it highlights an unusual intraoperative presentation of LV free wall rupture and the ideal circumstances for treating it.

## Introduction

Left ventricular (LV) free wall rupture is a frequently lethal complication of an acute myocardial infarction (MI) that usually occurs within three to seven days. It has been reported with an incidence as high as 7.6% among various populations of post-MI patients and, while occurrence is difficult to predict, it has been associated with female gender, increased age, hypertension, and lower glomerular filtration rate (GFR) [1,2]. Additionally, rupture appears to be primarily a complication of a patient’s first MI and is associated with less coronary narrowing than a fatal MI without rupture [3]. Fluids and inotropes are useful for initial management, but the only definitive treatment is surgical repair. A recent 2021 meta-analysis identified an operative mortality rate as high as 32% but separate studies have shown mortality rates up to 80% or higher if non-operative [4-6]. Here, we present the case of a ventricular free wall rupture that occurred in the operating room (OR) after the induction of general anesthesia and prior to the start of a planned coronary artery bypass graft (CABG).

## Case presentation

A 72-year-old woman with a history of hypertension, chronic obstructive pulmonary disease (COPD), and tobacco use presented to the hospital with substernal chest pain and was diagnosed with an inferior wall ST-segment elevation myocardial infarction (STEMI). A subsequent cardiac catheterization demonstrated 80% left main occlusion, 100% left circumflex occlusion, and up to 70% narrowing of the right coronary artery. An intra-aortic balloon pump (IABP) was placed during the cardiac catheterization and the patient was scheduled for a CABG procedure. She was also anticoagulated with a heparin infusion titrated to partial thromboplastin time (PTT). She required no inotropic or vasopressor support pre-operatively to maintain stable hemodynamics. 

On the morning of surgery, the patient was noted to be afebrile with a heart rate (HR) of 85 beats per minute, blood pressure (BP) of 138/57 mmHg, and oxygen saturation (SpO_2_) of 95%. Pre-operative labs were notable for a white blood cell count (WBC) of 19.2 (10^9^/L), hemoglobin 11.2 g/dl, hematocrit 34.3%, and PTT 58 seconds. Troponin values peaked and downtrended 24 hours prior to surgery at 24.7 ng/mL. All other labs were within normal limits, including electrolytes. 

Upon arrival to the OR, the patient was pre-oxygenated by face mask and induced with 8 mg of etomidate, 50 mg of 1% lidocaine, and 150 mcg of fentanyl. She was paralyzed with 50 mg of rocuronium. Intubation was uncomplicated with no hemodynamic perturbations. Subsequently, two large-bore peripheral intravenous (IV) cannulas were placed for access as well as an arterial line for close hemodynamic monitoring and frequent sampling of blood. 

Approximately seven minutes after induction, as the patient was being prepped for central line placement, the blood pressure acutely decreased with associated bradycardia. Central line placement was aborted to manage unstable hemodynamics and vasopressors were given without improvement. Three minutes after the initial drop in blood pressure, the surgical team was made aware and a transesophageal echocardiogram (TEE) probe was placed which showed tamponade with severe LV compression (Video 1). The patient arrested immediately after TEE probe placement. Subsequently, her chest was prepped with concurrent chest compressions and intermittent epinephrine boluses were given without effect. No central line was available as all resources were directed towards preparation for emergent cardiopulmonary bypass (CPB). 20,000 units of IV heparin was administered and the patient’s groin was cannulated for CPB. Upon rapidly opening the chest, ventricular rupture was seen in the inferior lateral wall (Video 2: Color Compare; Video 3). A double lumen central venous catheter (CVC) was then placed in the superior vena cava (SVC).

**Video 1:** Transgastric short-axis transesophageal echocardiogram (TEE) clip demonstrating a large pericardial effusion causing severe compression of the left ventricle.

**Video 2:** Transesophageal echocardiogram (TEE) video clip using color compare to demonstrate flow (right) through a defect in the inferolateral wall.

**Video 3:** A transgastric short-axis transesophageal echocardiogram (TEE) clip demonstrating the inferolateral wall defect.

The patient underwent primary repair of the free-wall rupture followed by a three-vessel CABG. The remaining procedure was uncomplicated with resolution of the hemopericardium (Video 4). Her initial postoperative course was complicated by ischemic changes requiring OR take-back to improve the flow of the saphenous vein graft (SVG) to the right coronary artery (RCA). Subsequently, the patient was noted to be neurologically intact with no deficits, and she was extubated successfully to room air. She was hospitalized for a total of 18 days and completed a course of subacute rehabilitation after discharge. 

**Video 4:** Post-procedure transesophageal echocardiogram (TEE) with displaying resolved hemopericardium

## Discussion

Intraoperative hypotension unresponsive to vasopressor administration demands a broad differential that must be acutely addressed by the perioperative physician. Generally accepted and known underlying causes include pneumothorax, cardiogenic shock, hypovolemia/hemorrhage, anaphylaxis, decompensated hypothyroidism, adrenal insufficiency, vasoplegic syndrome, and pre-operative angiotensin-converting enzyme inhibitor (ACE-I) usage. In this particular case, the patient was known to be euthyroid, had no symptoms of adrenal insufficiency or a history of chronic steroid use, and was not taking an ACE-I. Severe hypovolemia or hemorrhage seemed an unlikely cause given her clinical state at arrival to the OR. We quickly assessed the patient for signs of anaphylaxis but noted no visible rash or swelling, and no changes to her ventilator settings. Additionally, her lungs remained compliant when manually ventilated with no changes to her ventilation parameters or airway pressures, making obstructive lung pathology and pneumothorax less likely. At this point, cardiogenic shock was the most likely cause and was confirmed with tamponade on TEE.

Time to diagnosis and subsequent intervention is critical in this patient population. A clinical diagnosis of rupture will most likely include physical exam findings consistent with tamponade, such as hypotension, jugular venous distension (JVD), and pulsus paradoxus. Echocardiography remains the most critical tool for concrete diagnosis, and is associated with high sensitivity and specificity [7]. However, there have been recorded cases in which echocardiography was negative for rupture but surgical exploration did reveal the presence of a hole in the myocardium [8]. Of additional note, the question of pre-operative IABP placement is significant as prior studies have shown that it can reduce the transition from one type of rupture to another (oozing vs. blowout) as well as reduce the extension of the infarct and the chance of re-rupture if repaired [9,10]. However, these studies rely on very small sample sizes and a recent large meta-analysis failed to associate a protective effect with IABP placement, thus making its overall benefit unclear [4].

Once a free-wall rupture has been diagnosed, the first step is to maintain stable hemodynamics with fluids and inotropes, or to initiate advanced cardiac life support in the event of hemodynamic collapse [3,7]. In our case, the presenting sign of free-wall rupture was cardiovascular collapse with echo findings of tamponade. We provided vasopressor and inotropic support while chest compressions were initiated. The surgical team was able to rapidly prep the chest and peripherally cannulate in order to “crash” onto bypass.

We posit that this patient likely had the ideal circumstances for an event of this kind including a secure airway, invasive blood pressure monitoring, a perfusionist with a prepared CPB circuit, and a scrubbed surgical team. This situational placement is rarely available and management on the floor, emergency department, or intensive care unit (ICU) will be dependent on quick diagnosis and resource availability. Overall, this case illustrates the optimal conditions for patient survival and recovery following LV free wall rupture.

## Conclusions

This case highlights three pertinent learning points. First, refractory shock covers a broad differential. Intraoperatively, causes such as anaphylaxis, pneumothorax, and tamponade should be considered while also assessing for less substantial causes. Second, patients presenting for CABG within three to seven days after an MI are at risk for this fatal complication and their risk factors should be considered in the perioperative period. Third, and finally, once LV rupture is diagnosed, aggressive supportive care is essential but the only definitive treatment is surgical repair.
